# Relaxation of Shear-Induced Orientation and Textures in Semi-Dilute DNA Solutions

**DOI:** 10.3390/polym17182452

**Published:** 2025-09-10

**Authors:** Scarlett Elizabeth López-Alvarez, François Caton, Denis C. D. Roux, Félix Armando Soltero Martínez, Florian Scholkopf, Frédéric Nallet, Guillermo Toriz, Arnaud Saint-Jalmes, Marguerite Rinaudo, Lourdes Mónica Bravo-Anaya

**Affiliations:** 1 Doctorado en Ciencia de Materiales, División de Ingenierías, CUCEI, Blvd. M. García Barragán #1451, Universidad de Guadalajara, Guadalajara 44430, Jalisco, Mexico; scarlett.lopez@alumnos.udg.mx; 2 Institut des Sciences Chimiques de Rennes, Equipe CORINT, CNRS, UMR 6226, Campus de Beaulieu, Bat 10A, Université de Rennes, 35042 Rennes, France; 3 Institut de Physique de Rennes (IPR), CNRS, UMR 6251, 35000 Rennes, France; florian.scholkopf@univ-rennes.fr (F.S.); arnaud.saint-jalmes@univ-rennes.fr (A.S.-J.); 4 Laboratoire Rhéologie et Procédés, UMR CNRS 5520, Université Grenoble Alpes, Grenoble INP, 38000 Grenoble, France; francois.caton@univ-grenoble-alpes.fr (F.C.); denis.roux@univ-grenoble-alpes.fr (D.C.D.R.); 5 Departamento de Ingeniería Química, Universidad de Guadalajara, Blvd. M. García Barragán #1451, Guadalajara 44430, Jalisco, Mexico; j.soltero@academicos.udg.mx; 6 Centre de Recherche Paul Pascal, UMR5031-Université de Bordeaux, 33600 Pessac, France; frederic.nallet@u-bordeaux.fr; 7 Departamento de Madera, Celulosa y Papel, Universidad de Guadalajara, Km. 15.5 Carretera Guadalajara-Nogales, Zapopan 45110, Jalisco, Mexico; guillermo.tgonzalez@academicos.udg.mx; 8 Biomaterials Applications, 6 Rue Lesdiguières, 38000 Grenoble, France; marguerite.rinaudo38@gmail.com

**Keywords:** DNA solutions, textures, flow-birefringence, relaxation, anisotropy, rheo-SAXS

## Abstract

Recent studies on semi-dilute Calf-Thymus DNA (CT-DNA) solutions have revealed the presence of birefringence and small-scale textures influenced by shear and ionic strength. In this study, we investigate these phenomena on the same solutions to elucidate the underlying shear-induced supramolecular organization and relaxation dynamics using rheo-birefringence, rheology, and rheo-SAXS (small-angle X-ray scattering). Static SAXS confirmed concentration-dependent inter-chain correlations in the 15–25 nm range, while rheology revealed a slipping yield-stress behavior. Oscillatory strain sweep and steady state rheo-birefringence experiments correlated the appearance of textures with the onset of flow and a stress plateau observed over a shear rate range from approximately 1 to 1000 s^−1^. Transient rheo-birefringence and rheo-SAXS revealed two distinct relaxation mechanisms on well-separated time scales: a fast process lasting a few seconds, inversely proportional to the shear rate, consistent with the orientational relaxation of DNA segments on a ~20 nm scale; and a slower relaxation over tens of seconds, independent of the shear rate, associated with the disappearance of textures, and attributed to a diffusive process. These findings provide significant insights into the mechanisms governing DNA organization and dynamics in semi-dilute solutions under flow and highlight the need for temporally resolved start-up rheo-SAXS and rheo-birefringence measurements, as well as theoretical models describing these processes across various spatial and temporal scales.

## 1. Introduction

Nucleic acids, including deoxyribonucleic acid (DNA) and ribonucleic acid (RNA), are essential biomacromolecules found in all living organisms and play crucial roles in fundamental biological processes [1,2]. Nucleic acids are highly charged natural polyelectrolytes due to the phosphate groups present in each nucleotide [3,4]. Electrostatic interactions between charged DNA molecules and their counterion clouds significantly influence the key properties of DNA, such as its melting temperature, conformational changes (A, B, and Z forms), and persistence length [5,6]. Double-stranded DNA is a semi-flexible polymer with a high persistence length of approximately 50 nm (or 150 base pairs) in 0.1 M NaCl solution [7,8,9]. The overall shape and flexibility qualities of DNA are similar to those of an ideal very large linear polymer, making DNA one of the best physical representations of such polymers [10,11,12,13]. DNA solutions can exist in isotropic or anisotropic states and can form liquid crystalline phases [9,14,15].

Previous studies have investigated the physicochemical and rheological properties of calf-thymus DNA (CT-DNA) in Tris-HCl/EDTA (TE) buffer, at an estimated NaCl concentration of 10^−2^ M [3,16]. The molecular weight and polydispersity of CT-DNA have also been characterized in the literature, yielding values between 6 and 8.5 MDa [3,17,18,19]. The critical concentration C*, which marks the onset of physical interactions between individual polymer chains, was reported to be 0.24 mg/mL for CT-DNA [3,9]. Previous Small-Angle X-ray Scattering (SAXS) studies on CT-DNA solutions at 4 and 10 mg/mL revealed a well-defined correlation peak, reflecting long-range electrostatic repulsions between DNA chains and a characteristic preferential interchain distance [16]. Rheological measurements combined with optical observations under crossed polarizers in the 4–10 mg/mL range showed the appearance of a shear stress plateau, shear-induced birefringence, and small-scale textures (also known as meso-scale patterns, ~10–100 µm, observed under shear [20]). At high shear rates, these textures disappeared, and the solution exhibited homogeneous birefringence, indicating the full orientational alignment of the DNA molecules [9,16]. This behavior was influenced by the ionic concentration through screening electrostatic repulsions. The textures observed in 0.01 M NaCl were less pronounced than those in water, indicating a lower degree of chain organization. However, regardless of the ionic conditions, at higher shear, all CT-DNA chains aligned with the flow, resulting in birefringent solutions [16].

Despite these advances, several aspects remain to be elucidated. First, in the semi-dilute regime, the relationship between shear and structural organization across scales—from the molecular (~10 nm) to the macroscopic level —has yet to be determined. Second, previous studies in this concentration range have predominantly focused on the steady state characteristics of CT-DNA solutions, often accompanied only by qualitative birefringence measurements, which should be complemented with quantitative nanostructural data, such as Small-Angle X-ray Scattering (SAXS). In addition, the transient dynamics of DNA (dis)orientation under varying shear rates, as well as the mechanisms responsible for the formation or disappearance of textures, remain uncharacterized.

In this study, we investigate the shear-induced organization and relaxation dynamics of supramolecular structures in CT-DNA solutions within TE buffer at the nano-, meso-, and macro-scales, focusing on the same semi-dilute regime, in a concentration range of 7–16 mg/mL. SAXS measurements were first performed to assess the molecular interactions between DNA chains, revealed by correlation peaks indicating preferential interchain distances. Then, combined flow, start-up, and relaxation experiments were performed using in situ rheo-SAXS to monitor nano-scale structural changes, alongside optical rheo-birefringence to correlate macroscopic flow behavior with molecular orientation and texture evolution. Owing to constraints in experimental time acquisition, this study primarily characterizes the steady state behavior and the relaxation of supramolecular organization after shear cessation by simultaneously analyzing the birefringence, stress relaxation, and SAXS data.

## 2. Materials and Methods

### 2.1. Materials and Preparation of Solutions

Calf-Thymus DNA (CT-DNA), with a viscometric average molecular weight of 6,560,000 g/mol, was used for the preparation of solutions [3]. Deoxyribonucleic acid sodium salt from calf thymus (CT-DNA) used in our study was purchased from Sigma-Aldrich Chimie (catalog number D1501, Type I, fibers, CAS: 73049-39-5, Saint-Quentin-Fallavier, France). The CT-DNA sample was used as received, without additional chromatographic separation. The buffer solution was prepared by dissolving Trizma (Tris–HCl, C_4_H_12_ClNO_3_) at 100 mM and ethylenediaminetetraacetic acid (EDTA, C_10_H_16_N_2_O_8_) at 10 mM, both with a purity of 99.0% added. EDTA chelates divalent metal ions, thus preventing DNAse-mediated degradation [21]. Under these conditions, DNA degradation is unlikely [22]. The buffer solution was adjusted with 3 M NaOH at pH = 7.4. For preparation of CT-DNA solutions with better stability, the buffer solution was used after dilution with HPLC water to a 9:1 ratio and named TE buffer, with an ionic concentration equivalent to 10 mM NaCl [3]. To prevent evaporation and potential concentration changes, the vials were sealed with Parafilm^®^ (Neenah, WI, USA). CT-DNA/TE samples were stored at 4 °C in a refrigerator and used for experiments after one week to achieve good solubility. All the reagents were supplied by Sigma-Aldrich Chimie (Saint-Quentin-Fallavier, France).

### 2.2. Small-Angle X-Ray Scattering Measurements

SAXS experiments of CT-DNA/TE solutions were performed on ID02 high brilliance beamline at European Synchrotron Radiation Facility (ESRF, Grenoble, France) [23]. DNA solutions were placed in a quartz capillary of a flow-through cell (2 mm path length). The experimental setup utilized a wavelength of λ = approximately 0.1 nm (energy of 12.23 keV, with a two-dimensional Eiger 4M detector (Dectris, Baden-Dättwil, Switzerland) at sample-to-detector distances positioned at 1.5 m, covering a q-range of 0.05–5 nm^−1^, where q = (4π/λ) sin(θ/2), with θ the scattering angle and λ the wavelength [24]. SAXS measurements were performed at 20 °C. Background correction was applied by subtracting solvent scattering, and the data were processed with SAXS utilities software, version 1.024 [25].

### 2.3. Rheological Measurements

The rheological behavior of CT-DNA/TE buffer solutions was studied in a concentration range between 7 and 16 mg/mL using dynamic, transient and flow measurements with a MCR Physica 301 stress-controlled rheometer from Anton Paar (Les Ulis, France). All of these measurements were made at 20 °C with a steel cone geometry with a diameter of 40 mm and an angle of 1.992°.

*(a)*  *Dynamic measurements*: Strain measurements were performed to determine the behavior of CT-DNA/TE buffer solutions in the linear domain. The linear viscoelastic regime (LVR) was determined by performing strain sweeps at an angular frequency of 1 Hz, with strain values ranging from 0.1% to 500% and data points recorded every 10 s.
*(b)*  *Steady state flow measurements:* Upward and downward measurements were performed over a shear rate range from 10^−3^ to 1000 s^−1^. At each selected shear rate, the sample was allowed to flow until the measured shear stress reached a constant value, indicating that the stationary state had been reached.
*(c)*  *Transient measurements:* The transient shear stress behavior was evaluated by applying shear rates ranging from 10^−2^ to 1000 s^−1^, with measurement duration of at least 100 s, depending on the applied shear rate.
*(d)*  *Relaxation measurements:* To evaluate the relaxation behavior of CT-DNA/TE buffer solutions, shear rates ranging from 0.005 to 1000 s^−1^ were applied for at least 100 s, depending on the shear rate. After stopping the shear, the subsequent decrease in shear stress was recorded over a 400 s period.

### 2.4. Visual Observations Under Crossed-Polarizers

**Figure 1:**
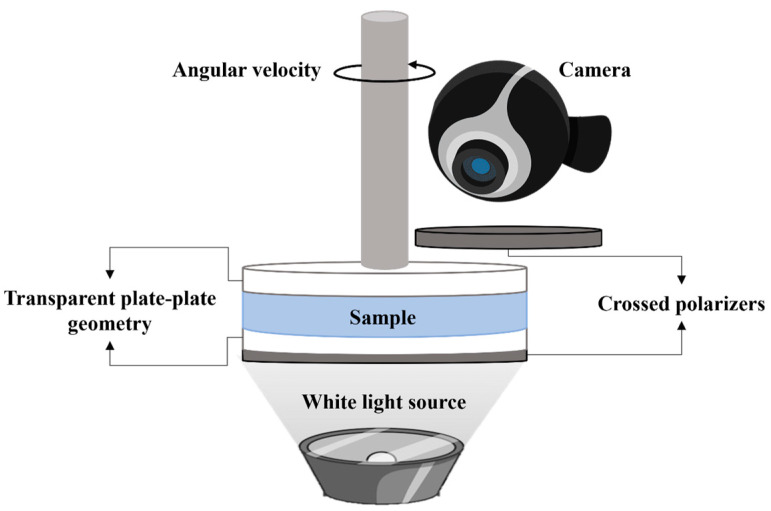
Schematic representation of the setup coupled to the Anton Paar Physica MCR 301 rheometer for observing the birefringence and texture formation under shear.

Birefringence was observed using a Physica MCR 301 rheometer from Anton Paar, equipped with a 43 mm diameter glass plate/plate geometry. The gap between the plates was set to 0.65 mm. The plates were positioned between two crossed polarizers, one placed below the fixed plate and the other one above the geometry (Figure 1). The sample was illuminated vertically with a white light source positioned beneath the first polarizer. Images of CT-DNA/TE buffer solutions under shear and between crossed-polarizers are extracted from recorded videos taken during transient and relaxation measurements at different shear rates (from 0.005 to 1000 s^−1^) using a Logitech network camera connected directly to the computer. Visual observations were taken at 20 °C.

### 2.5. Small-Angle X-Ray Scattering Measurements Coupled to Rheology

**Figure 2:**
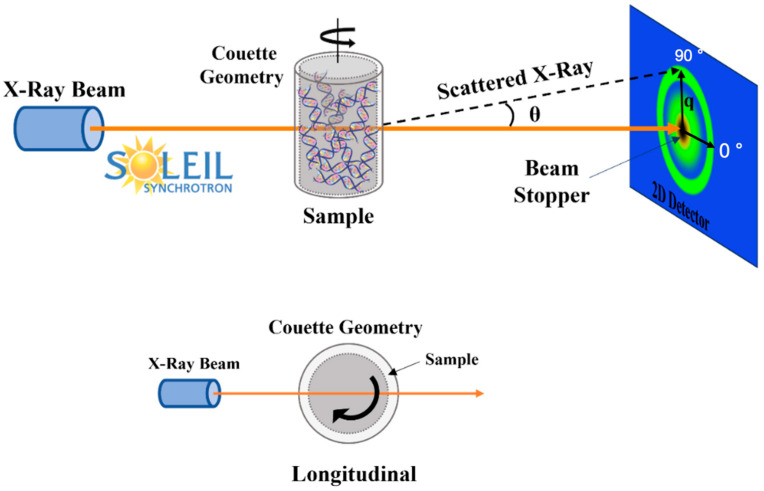
Rheo-SAXS setup and observation planes related to longitudinal beam position. The orange arrows correspond to the X-ray beam and the green rings to the scattering diffusion patterns.

Rheo-SAXS experiments, combining rheology with SAXS, were performed at the SWING beamline of the SOLEIL synchrotron in Gif-sur-Yvette, France, for CT-DNA samples at concentrations of 10 and 15 mg/mL using water as solvent. Sample shearing was precisely synchronized with SAXS data acquisition using an Anton Paar MCR 501 controlled stress rheometer. CT-DNA/TE samples were loaded into a polycarbonate Couette cell (Anton Paar) featuring a 500 µm gap, an internal diameter of 20 mm and a height of 17 mm, optimized for efficient X-ray transmission [26]. Flow measurements were performed by applying shear rates of 1, 10, 100, and 1000 s^−1^ for a duration of approximately 100 s and 0.1 s^−1^ for around 200 s, followed by shear cessation. SAXS data were collected at 12 keV (λ ~ 0.1 nm) with a fixed sample-to-detector distance of 1 m using an Eiger X 4M detector (Dectris). For each applied shear rate or strain, 100 two-dimensional SAXS frames were recorded with an exposure time of 200 ms per frame at a frame rate of one image every 3 s. Only longitudinal scattering results (that is to say: X-ray incident beam crossing the Couette rotation axis) at 0° and 90° (2D frames masked by 20° wide angular sectors along the detector axes perpendicular and parallel to the Couette rotation axis, respectively) are presented here (Figure 2). The solvent background was subtracted from the raw data, and the resulting patterns were processed using Foxtrot 3.5 (collaboration between XENOCS, Grenoble, France, and the SOLEIL synchrotron SWING beamline team) and SAXS utilities software.

## 3. Results and Discussion

### 3.1. Steady State Measurements

#### 3.1.1. Organization of CT-DNA/TE Solutions by SAXS Measurements

SAXS measurements were conducted on CT-DNA/TE solutions prepared at concentrations ranging from 7 to 16 mg/mL.

**Figure 3:**
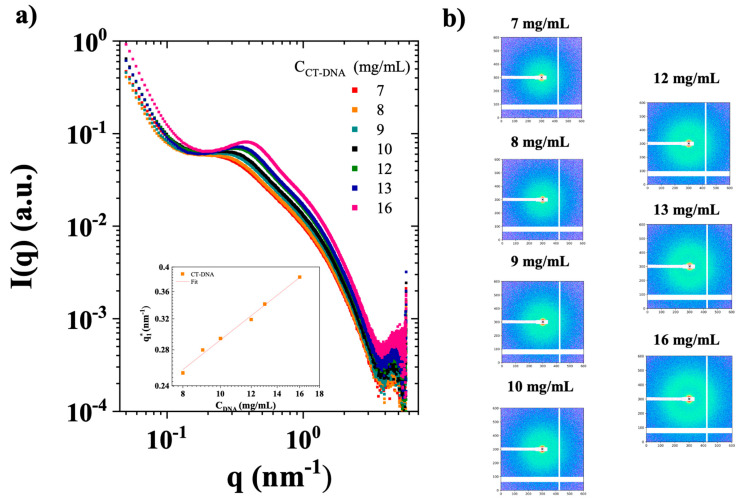
(**a**) Small-angle X-ray scattering (SAXS) intensity I(q) (arbitrary units, a.u.) as a function of the scattering vector (q) for CT-DNA solutions in TE buffer at the concentrations of 7–16 mg/mL, measured at 20 °C. The inset shows the evolution of the maximum value of q_1_* as a function of polymer concentration in double logarithmic scales. (**b**) Corresponding 2D SAXS patterns. Samples are plotted with square pixels, with log intensity scales shown.

Figure 3a shows the angle averaged SAXS intensity I(q) for these samples while Figure 3b) shows the 2D SAXS patterns. Firstly, the 2D patterns show the progressive appearance of a ring with an isotropic scattering. Secondly, a concentration-dependent correlation peak was observed at q_1_* from ~0.25 to 0.4 nm^−1^ on the angle averaged intensities, corresponding to a spacing distance (d) of approximately 15 to 25 nm (d = 2π/q_1_*) [27]. This feature, commonly seen in polyelectrolyte solutions, reflects the average CT-DNA inter-spacing, governed by dilution owing to electrostatic repulsions between polymer chains [28]. Similar effects have been previously reported in light and neutron scattering experiments [16,29,30,31]. The inset in Figure 3a presents the evolution of q_1_* as a function of CT-DNA concentration, which closely follows the scaling law of q_1_*~C^1/2^, consistent with previous studies [16,28].

#### 3.1.2. Qualitative Flow Birefringence

CT-DNA/TE solutions with concentrations ranging from 7 to 16 mg/mL were examined between crossed polarizers while applying a small, gravity-induced flow by tilting the container at a large angle.

**Figure 4:**
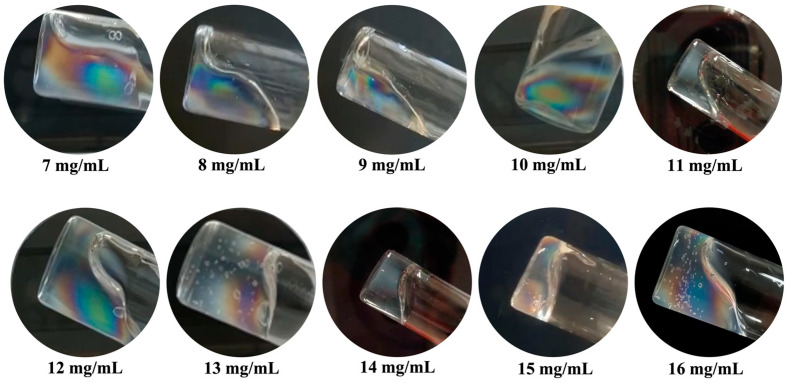
Images of CT-DNA/ TE buffer at C_DNA_ from 7 to 16 mg/mL and stored in vials (inner diameters in the range of 2 cm) observed under gentle flow between crossed polarizers. Observations were carried on at room temperature.

In Figure 4, all CT-DNA/TE samples exhibited two interesting properties. First, the interfaces are very different from those that would be observed in fluids in a similar experiment, bubbles included, suggesting that all those materials behave as yield stress fluids, in agreement with previous work [16]. Second, in all experiments, colored patterns are observed, characteristic of flow birefringence. In addition, birefringence vanished quite rapidly upon cessation of the flow, suggesting relaxation back to the apparently optically isotropic state.

#### 3.1.3. Steady State, Viscoelastic and Birefringence Properties

**Figure 5:**
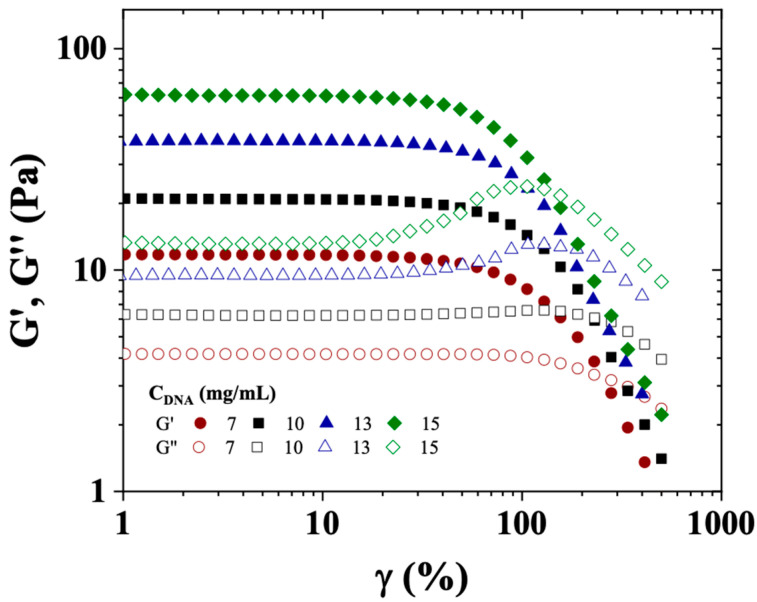
Elastic modulus (G′, solid symbols) and viscous modulus (G″, open symbols) as a function of strain (γ(%)) for CT-DNA/TE solutions at 7, 10, 13, and 15 mg/mL. Measurements were conducted at a temperature of 20 °C, a frequency of 1 Hz, and using a cone/plate geometry.

The rheological behavior of the CT-DNA/TE solutions in the semi-dilute regime was studied through dynamic and flow measurements. Deformation sweeps oscillatory measurements were performed at different CT-DNA concentrations from 7 to 15 mg/mL, to determine the linear viscoelastic region (LVR), which corresponds to the region where the elastic and viscous moduli (G′, G″, respectively) are independent of the deformation (γ) (Figure 5).

Figure 5 shows the dependence of G′ and G″ on γ (%) for CT-DNA solutions prepared at concentrations of 7, 10, 13 and 15 mg/mL, and analyzed at 20 °C. All CT-DNA solutions showed a wide linear viscoelastic region, up to around 35, 30, 28 and 22% of strain, for concentrations of 7, 10, 13 and 15 mg/mL, respectively. Within the restricted concentration range used in this work, the elastic modulus followed a power law as a function of polymer concentration (C) as follows: G′~C^2.1^, in good agreement with the experimental value given by Mason et al. (2.3) and the predicted exponent (9/4) in good solvent by de Gennes [32,33].

**Figure 6:**
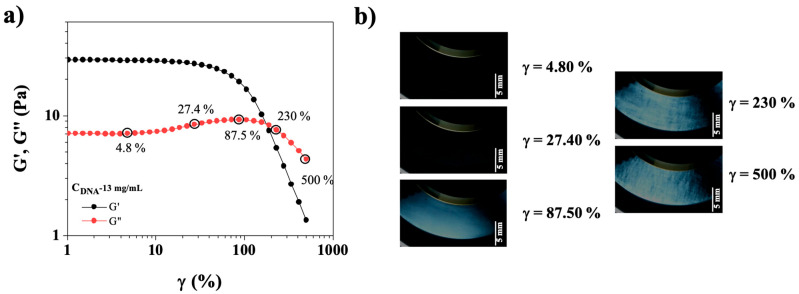
(**a**) Elastic modulus (G′, black symbols) and viscous modulus (G″, red symbols) as a function of strain (γ (%)) for CT-DNA/TE solution at 13 mg/mL. Measurements were carried out at 20 °C and frequency 1 Hz. (**b**) Photographs of DNA solution, 13 mg/mL, were taken between crossed polarizers at γ = 4.80, 27.40, 87. 50, 230, and 500%, scale bar is equal to 5 mm. Plate/plate geometry with an observation gap of 0.65 mm (cf. Figure 1).

The evolution of the appearance of CT-DNA/TE solutions as a function of the deformation was examined for several DNA concentrations under crossed-polarizers. An example of the structural evolution of the CT-DNA/TE solutions is shown for the 13 mg/mL concentration in Figure 6.

Figure 6a shows the elastic and viscous moduli (G′, G″, respectively) for CT-DNA/TE solutions at 13 mg/mL in oscillatory strain sweep experiment performed simultaneously with the birefringence observations. Birefringence was observed at 87.50%, when the CT-DNA solution exits the linear viscoelastic regime (Figure 6b). Following the bump very often observed in yield stress fluids, both G′ and G″ cross and sharply decreased, defining a yield point. Small-scale (sub-millimeter) textures appeared in this region.

**Figure 7:**
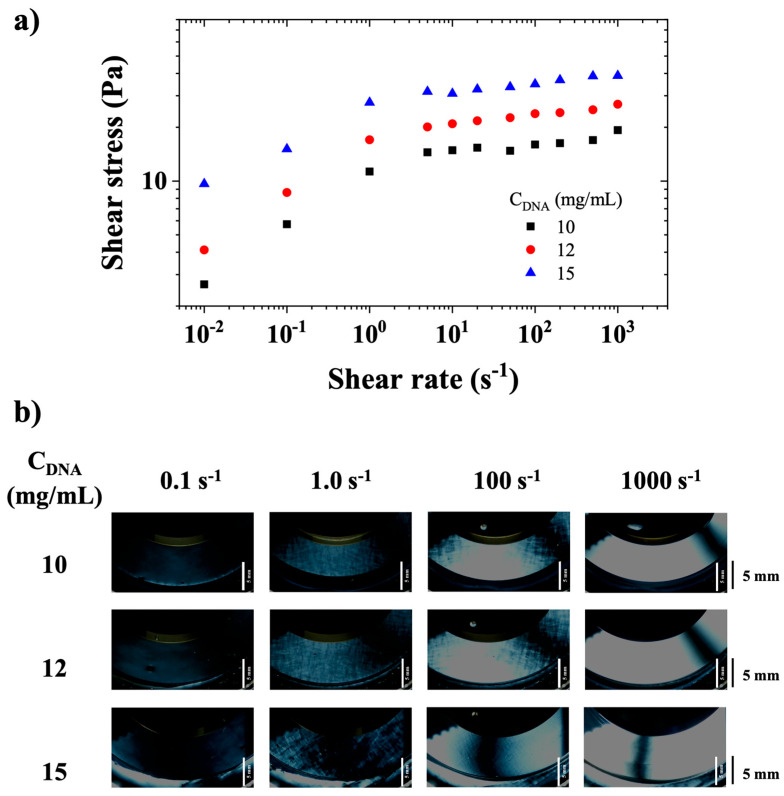
(**a**) Shear stress as a function of shear rate for CT-DNA/TE solutions with concentrations of 10, 12, and 15 mg/mL. (**b**) Visualizations of DNA solutions under stationary flow observed between crossed polarizers for CT-DNA/TE solutions prepared at the same concentrations. Observation with a gap = 0.65 mm in a plate/plate geometry (cf. Figure 1). The bar on the right represents a distance of 5 mm. Measurements were performed at 20 °C.

Figure 7a shows the steady state flow curves of CT-DNA solutions along with birefringence observations under crossed polarizers (Figure 7b) at selected shear rates (0.1, 1, 100, and 1000 s^−1^) and concentrations of 10, 12, and 15 mg/mL.

Figure 7a shows a rheological behavior that can be described as a slipping yield stress fluid, in agreement with the visual observations of the previous section and literature [16]. CT-DNA/TE solutions appear to slip at walls below 1 s^−1^, and then presents a stress plateau that extends from approximately 1 s^−1^ to 1000 s^−1^. Figure 7b shows the appearance of birefringence at a shear rate of about 0.1 s^−1^, then of small-scale textures at approximately 1 s^−1^, and homogeneous birefringence at 1000 s^−1^.

#### 3.1.4. Steady State Rheo-SAXS

**Figure 8:**
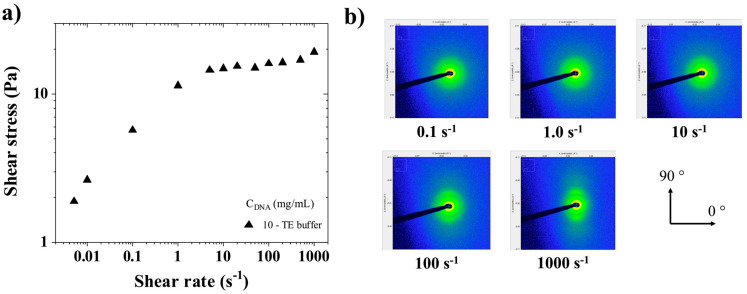
(**a**) Shear stress (σ) as a function of shear rate $\dot{\gamma }$ for CT-DNA/TE solutions at 10 mg/mL. Measurements were performed at 20 °C. (**b**) Sequential time-resolved SAXS images taken in the longitudinal configuration at different shear rates in the steady state (0.1, 1.0, 10, 100, and 1000 s^−1^).

Figure 8 illustrates the shear-induced structural evolution of CT-DNA solutions through combined rheological and SAXS measurements.

Rheo-SAXS measurements were performed at two concentrations (10 and 15 mg/mL), as described in Section 2.5. Sequential SAXS images were recorded every 3 s in the longitudinal setup (cf. Figure 2), where the X-ray beam was perpendicular to the rotation axis of the flow cell. Figure 8a shows the flow curve of shear stress versus the shear rate for CT-DNA/TE solutions at 10 mg/mL. Figure 8b shows sequential time-resolved SAXS patterns collected in the longitudinal configuration.

**Figure 9:**
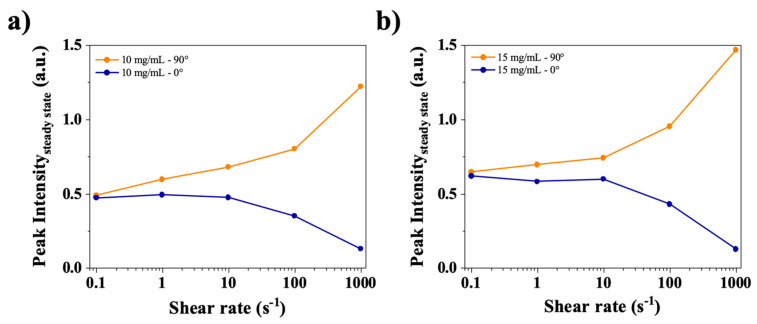
Steady state intensities at the peak q_1_* (arbitrary units, a.u.) for the 0° and 90° directions for (**a**) 10 mg/mL and (**b**) 15 mg/mL in water.

Because anisotropy is observed in the SAXS images, it is interesting to plot the steady state peak intensity in both the horizontal and vertical axes (resp. 0° and 90°, cf. Figure 2). In Figure 9, for both 10 and 15 mg/mL solutions, the intensities at 0.1 s^−1^, 0° and 90° are the same and start to diverge at 1 s^−1^, in the same region where the yield stress fluid appears to stop slipping. In other words, below 1 s^−1^, the yield stress fluid exhibits slip, with most of the shear occurring in the slip layers. Hence there is little to no birefringence because the bulk of the material is weakly sheared. Then, when increasing the shear rate above 1 s^−1^, the slip becomes negligible and the yield stress material flows showing flow birefringence as well as textures [34,35].

To summarize the steady state findings, the oscillatory and shear flow measurements, the presence of large bubbles in the sample as well as the qualitative behavior of the free surface when tilting the vials, all point to a yield stress fluid rheological behavior, with slipping at walls below shear rates of about 1 s^−1^, in agreement with previous findings [16]. At the same time, under negligible slipping conditions, all samples showed both strong flow birefringence and SAXS anisotropy, demonstrating at least a partial average orientation of CT-DNA molecules. In the same conditions, textures and a pronounced q_1_* peak were also evident suggesting a link between those two observations.

### 3.2. Relaxation of CT-DNA Solutions After Flow Shearing

To understand the relaxation mechanisms of CT-DNA/TE solutions submitted to imposed shear, the relaxation of shear stress, birefringence, and SAXS intensity were measured after stopping the applied shear rates. For each technique, the relaxation time (τ) was defined as the time required for the corresponding normalized signal to decrease to half of its initial steady state value.

#### 3.2.1. Rheological Measurements Coupled with Birefringent Observations

**Figure 10:**
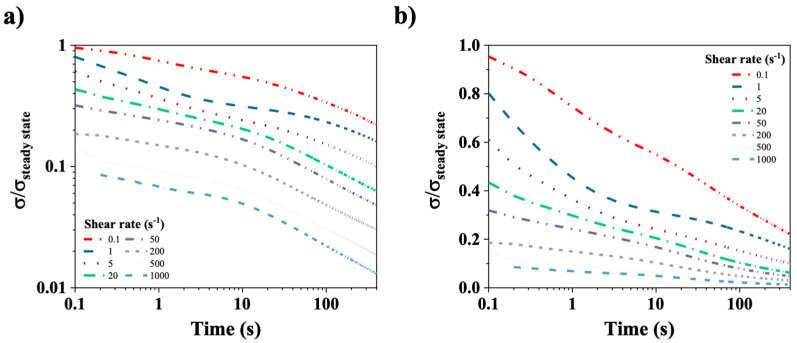
(**a**) Normalized shear stress (σ/σ_steady state_) relaxation curves on a log-log scale. (**b**) Normalized shear stress (σ/σ_steady state_) relaxation on a log-lin scale. The time zero corresponds to the stopping time of the shear and time is displayed in a logarithm scale. CT-DNA/TE solution at 15 mg/mL was used in these experiments. Measurements were performed at 20 °C.

Figure 10a,b show the relaxation of the normalized shear stress (σ/σ_steady state_) for a CT-DNA/TE solution at 15 mg/mL plotted on log-log (left) and log-lin (right) scales. In this experiment, shear rates in the range from 0.1 to 1000 s^−1^ were applied to the CT-DNA/TE solutions until the shear stress reached a steady state. The shear rate was abruptly stopped at t = 0. Subsequent relaxation of the CT-DNA/TE solution was monitored for approximately 400 s. The curves at 10 and 12 mg/mL are very similar. 

**Figure 11:**
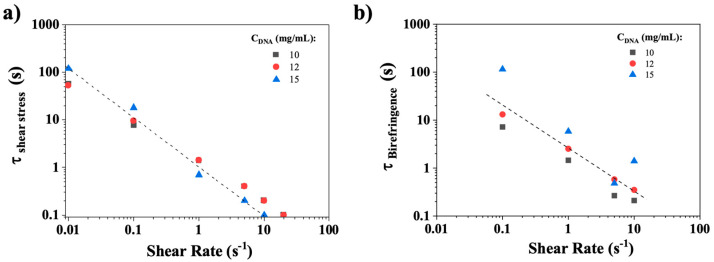
Relaxation times as a function of shear rate imposed for 10, 12, and 15 mg/mL CT-DNA/TE samples obtained from (**a**) shear stress rheology and (**b**) birefringence determination. The dashed lines represent power laws with a −1 slope. Measurements were conducted at 20 °C.

Figure 10a,b illustrate complex temporal behaviors that present challenges for quantitative analysis. Figure 10b clearly demonstrates a rapid relaxation time, in contrast to Figure 10a, which evidences a slower, power-law relaxation mechanism. This indicates the involvement of at least two processes in stress relaxation. It is important to note that only the fast stress relaxation times could be evaluated for shear rates smaller than 20 s^−1^, as the initial normalized stress point in time is less than 0.5. Indeed, as previously mentioned, the relaxation time (τ) was determined as the time needed for the normalized shear stress to decrease to half its steady state value. The values determined for CT-DNA concentrations at 10, 12 and 15 mg/mL and shear rates from 0.01 to 20 s^−1^ are presented in Figure 11a.

Figure 11a illustrates the rapid relaxation times of shear stress as a function of the imposed shear rate for varying concentrations of CT-DNA (10, 12, and 15 mg/mL). The rapid stress relaxation time follows a power law with a slope approximately equal to −1, as indicated by the dotted line. A similar behavior was observed in the birefringence relaxation times (Figure 11b). This suggests that the product of the characteristic time $\left(\tau_{c}\right)$ and the shear rate remains nearly constant and close to 1: $\tau_{c}\dot{\gamma }∼1$, a relationship previously observed in the orientational relaxation of polymeric liquid crystals [36,37,38], suggesting the involvement of similar mechanisms.

#### 3.2.2. Qualitative Birefringence Relaxation

**Figure 12:**
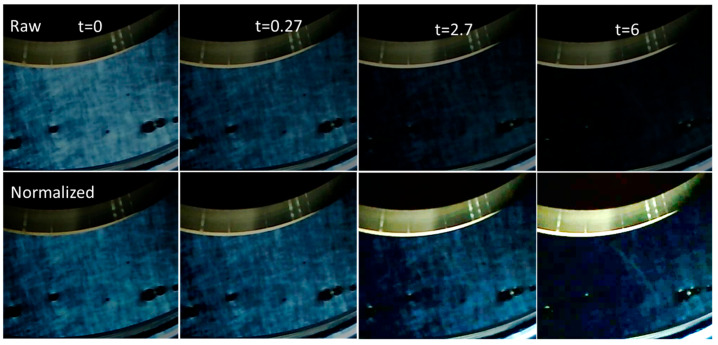
Example of birefringence relaxation after stopping shear (10 s^−1^) for CT-DNA/TE solution at 10 mg/mL. **Top**: Images extracted from the movies. **Bottom sequence**: The same images, but with the intensity normalized to the second image to obtain the best contrast. Measurements were performed at 20 °C.

Before showing the quantitative birefringence relaxation measurement results, it is interesting to visually observe birefringence relaxation. The images shown in Figure 12 were taken at chosen times during the birefringence relaxation after stopping the shear (steady state obtained at 10 s^−1^) for a CT-DNA/TE concentration of 10 mg/mL.

The top row shows the raw images extracted directly from the movie (see Video S1), indicating that birefringence relaxation occurs on a time scale of a few seconds under these conditions. Furthermore, the textures are clearly visible and persist. To verify this possibility, the same image sequence was intensity-normalized versus the second image (t = 0.27 s), which produced the best contrast and readability (Figure 12, bottom). In this sequence, the textures did not change in appearance, suggesting that the relaxation time scale of the textures was much larger than that of the birefringence.

**Figure 13:**
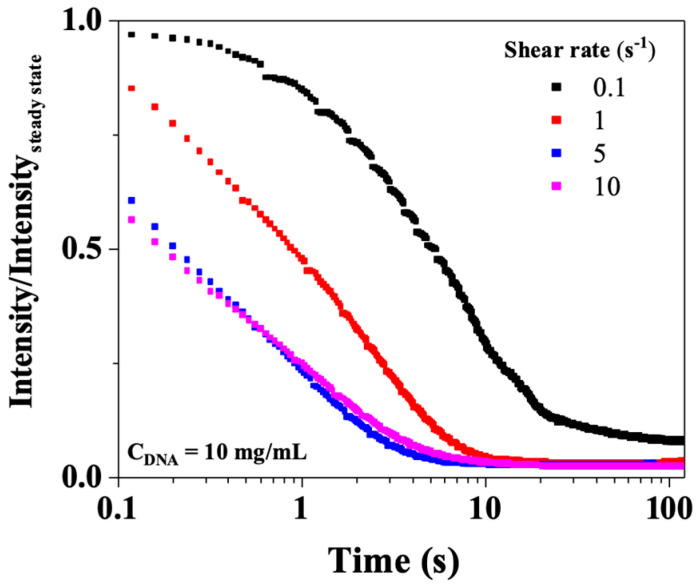
Birefringence relaxation curves after stopping shear (0.1, 1, 5, and 10 s^−1^) for CT-DNA/TE solution at 10 mg/mL. Measurements were performed at 20 °C.

Figure 13 shows, for CT-DNA/TE solution at 10 mg/mL, the birefringence relaxation curves (Intensity/Intensity_steady state_) recorded for shear rates between 0.1 and 10 s^−1^, the images being recorded at a frame rate of 15 Hz. For all the CT-DNA concentrations, the birefringence relaxation curves are much simpler than the corresponding shear stress curves, suggesting that a single process is responsible for relaxation. Figure 11b shows the birefringence relaxation times plotted as a function of the shear rate for 10, 12, and 15 mg/mL. The relaxation times decreased with the shear rate values, and a power law similar to that observed in the shear stress relaxation. This strongly suggests that the fast rheological relaxation time corresponds to the birefringence relaxation time, that is, the orientational relaxation of CT-DNA.

#### 3.2.3. Structural Relaxation of CT-DNA Solutions Observed by Rheo-SAXS Measurements

**Figure 14:**
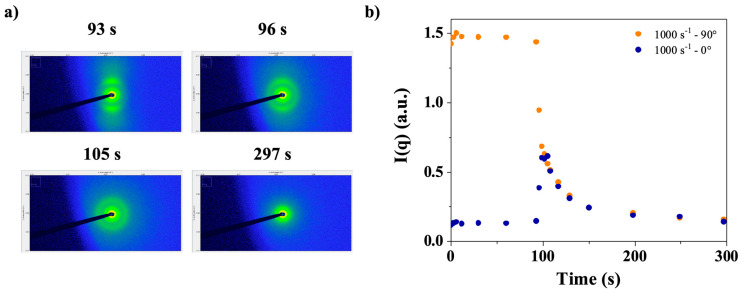
(**a**) Sequential SAXS patterns obtained in the longitudinal configuration (horizontal X-ray beam perpendicular to the vertical rotation axis of the flow cell) indicating that 93 s is enough for reaching steady state and immediately after shear stopping (96–297 s). (**b**) Peak intensity, q_1_*, at 0° and 90° as a function of time, measured at steady shear rate of 1000 s^−1^ and after shear cessation. CT-DNA in water at 15 mg/mL was used in these experiments. Measurements were performed at room temperature.

Figure 14a displays SAXS images taken at steady state (93 s) and a shear rate of 1000 s^−1^, immediately after stopping the shear in a CT-DNA solution at a concentration of 15 mg/mL in water. As shown previously [16], the behavior of DNA in water and TE buffer is nearly identical. Figure 14b shows the analysis of the intensity’s evolution as a function of time. Two main observations can be made from these images. First, the image taken during steady state shear (93 s) shows obvious anisotropy (Figure 14a), as pointed out in Section 3.1.4. Then, in the images taken at 96 and 105 s a ring was observed, which corresponded to the q_1_* peak. To characterize both the anisotropy and q_1_* peak relaxation, the intensity at the peak was plotted at 0° (horizontal) and 90° (vertical) (cf. Figure 2), as shown in Figure 14b.

Figure S1 provides an example of the data treatment used to extract intensity values, showing the time-dependent evolution of the sector-masked, azimuthally averaged scattered intensity of a 15 mg/mL CT-DNA solution in water upon application of a shear rate of 1000 s^−1^ followed by shear cessation. The intensity was taken at the peak maximum. The steady state is plain to see, with a large difference in intensity between the two 0–90° directions. When shear was stopped, a sudden decrease in I_90°_ and a sudden increase in I_0°_ were observed. Then, both curves merge, and the peak is not yet relaxed, whereas the anisotropy is.

**Figure 15:**
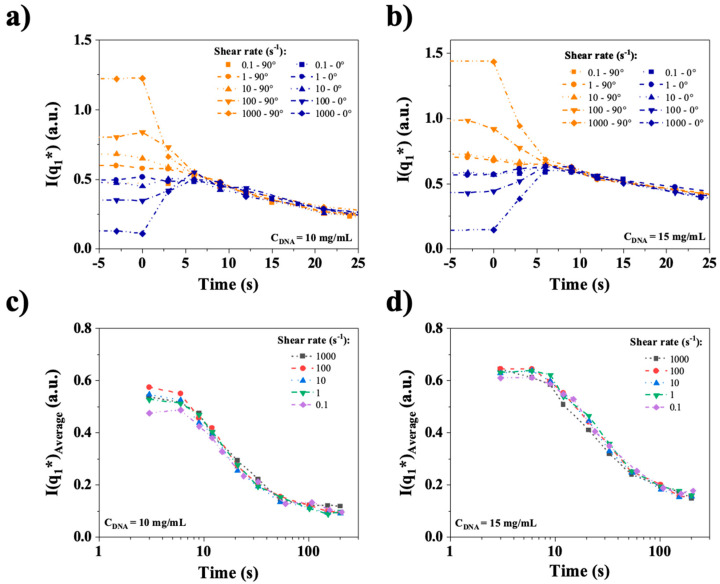
Top (**a**) and (**b**): Temporal relaxation of I_0°_ and I_90_° at shear rates ranging from 0.1 to 1000 s^−1^. Bottom (**c**) and (**d**): Relaxation of the isotropic peak intensity, calculated as (I_0°_ + I_90°_)/2. CT-DNA in water at 10 mg/mL and 15 mg/mL. All the measurements were performed at room temperature.

To better highlight this complex behavior, the temporal relaxations of I_0°_ and I_90°_ are plotted again in Figure 15a,b for the different imposed shear rates. Once the shear is stopped, indicated by time = 0, the relaxation starts, and the anisotropy decreases rapidly. All the curves of both figures collapse on a single curve for times larger than approximately 5–10 s.

This means that anisotropy decreases on time scales similar or faster than the image acquisition rate of the experiment (one image every 3 s), and that after a few seconds, the decay of an isotropic organization, related to textures, is observed, which occurs on a much longer time scale. To investigate the potential shear rate dependence of this isotropic relaxation of the main peak, the quantity (I_0°_ + I_90°_)/2, which is the isotropic SAXS intensity, is plotted versus time in Figure 15c,d. Remarkably, no shear dependence of the relaxation of this organization was observed at either 10 or 15 mg/mL, indicating a strictly diffusive behavior. Moreover, the relaxation time scales were 20 and 40 s for 10 and 15 mg/mL, respectively, which are compatible with the long relaxation times observed in rheometry, as well as the persistence of textures in the qualitative birefringence relaxation experiment.

The first takeaway of this relaxation study is that rheometry, birefringence, and SAXS measurements coherently show that the orientation relaxes rapidly on time scales of a few seconds. Furthermore, this characteristic relaxation time evolves inversely proportional to the applied shear rate, in a similar way to that of polymeric liquid crystals [36,37,38]. Recalling that the spatial scale of the SAXS measurements is considerably smaller than those of rheometry and birefringence (~20 nm vs. millimeters or centimeters), and given the very large molar mass of the CT-DNA molecule (~6 MDa), this suggests that it is not the whole molecule that aligns, but segments of size corresponding to the persistence length which number progressively increases as shear rate increases. Concerning textures relaxation, the qualitative birefringence observations suggested much larger relaxation times compared to orientation. This was confirmed and quantified using Rheo-SAXS which showed that the isotropic spatial structure (q_1_* peak) relaxed on time scales of several tens of seconds. Remarkably, this slow relaxation proved to be independent of the shear rate, pointing at a diffusive process that relaxes textures. This suggests that texture could be seen as local concentration variations.

## 4. Conclusions

Small-angle X-ray scattering (SAXS) confirmed electrostatic correlations and structural organization at q_1_* for CT-DNA/TE solutions in the concentration range of 7–16 mg/mL, which confirms their polyelectrolyte nature. As CT-DNA concentration increased, q_1_* sharpened and shifted to higher *q*-values, showing a decrease in the average interchain distance, consistent with the expected scaling law.

Under steady shear flow, all samples showed both pronounced flow birefringence and SAXS anisotropy above 1 s^−1^, when the threshold shear rate where those yield stress fluids stopped slipping, as evidenced by the steady state flow curves. The excellent correlation between birefringence and SAXS anisotropy demonstrated the existence of an average orientation of the semi-flexible CT-DNA molecules. This orientation relaxes rapidly on time scales of a few seconds which is inversely proportional to the applied shear rate, similar to polymeric liquid crystals, likely involving the same underlying mechanisms. The fast relaxation for a CT-DNA solution of very large molecules, as well as the fact that the spatial scale of the SAXS measurements is considerably smaller than those of rheometry and birefringence (~20 nm vs. millimeters), show that it is not the whole molecule that aligns, but segments of sizes corresponding to the persistence length.

At sufficiently high shear rates, distinct textures were identified using qualitative birefringence observations. Those textures exhibit relaxation times much longer than that of birrefringence associated with orientational relaxation. Rheo-SAXS analysis further revealed a spatial structuration, indicated by the q_1_* peak superimposed on the anisotropy. This structure relaxed over time scales of several tens of seconds, which was again considerably slower than the orientation relaxation. Notably, this slow relaxation was found to be independent of the shear rate, suggesting a diffusive process likely relaxing local concentration variations.

The present findings offer significant insights into the mechanisms governing DNA organization and dynamics in semi-dilute solutions under flow conditions. The temporal resolution of SAXS and birefringence measurements, combined with gap-dependent rheo-SAXS experiments at intermediate scales, will be essential for gaining a more thorough understanding of texture formation and its relationship with concentration variations, as well as the potential influence of slip. Furthermore, future research should focus on developing theoretical models capable of describing these processes across various spatial and temporal scales.

## Data Availability

The original contributions presented in this study are included in the article/Supplementary Materials. Further inquiries can be directed to the corresponding author.

## Supplementary Materials

The following supporting information can be downloaded at: https://www.mdpi.com/article/10.3390/polym17182452/s1. Figure S1. Intensity profiles from rheo-SAXS measurements at selected time intervals, illustrating the alignment during shear (1000 s^−1^) and subsequent relaxation of DNA chains in the longitudinal configuration at (a) 90° and (b) 0°. CT-DNA solution was prepared at 15 mg/mL in water. Measurements were carried on at room temperature. Video S1: Video of a DNA solution prepared at 10 mg/mL under flow and during relaxation after stopping shear (10 s^−1^). Observation with a gap = 0.65 mm in a plate-plate rheometer (cf. Figure 1). Measurements were performed at a temperature of 20 °C.
