# Supplementary material for: Relaxation of Shear-Induced Orientation and Textures in Semi-Dilute DNA Solutions
Source: Polymers (Basel). 2025 Sep 10;17(18):2452. doi: 10.3390/polym17182452 (PMC12473322; doi:10.3390/polym17182452)
Supplement: Supplementary file 1 [file polymers-17-02452-s001.zip › Supplementary information final.pdf]

Supplementary information

Relaxation of shear-induced orientation and textures in semi-dilute DNA solutions

Scarlett Elizabeth López-Álvarez[1,2,3], François Caton[4], Denis C.D. Roux[4], J. Félix Armando Soltero Martínez[5], Florian Scholkopf[3], Frédéric Nallet[6], Guillermo Toriz[7], Arnaud Saint-Jalmes[3], Marguerite Rinaudo[8], Lourdes Mónica Bravo-Anaya[2*]

[1] Universidad de Guadalajara, Doctorado en Ciencia de Materiales, División de Ingenierías, CUCEI, Blvd. M. García Barragán #1451, C. P. 44430, Guadalajara, Jalisco, México; scarlett.lopez@alumnos.udg.mx

[2] Université de Rennes, Institut des Sciences Chimiques de Rennes, équipe CORINT, CNRS, UMR 6226, Campus de Beaulieu, Bat 10A, 35042, Rennes Cedex, France; lourdes-monica.anaya@univ-rennes1.fr

[3] Université de Rennes, CNRS, IPR, Institut de Physique de Rennes, UMR 6251, F-35000, Rennes, France; florian.scholkopf@univ-rennes.fr, arnaud.saint-jalmes@univ-rennes.fr

[4] Université Grenoble Alpes, CNRS, Grenoble INP• 38000 Grenoble, France; denis.roux@univ-grenoble-alpes.fr, francois.caton@univ-grenoble-alpes.fr

[5] Universidad de Guadalajara, Departamento de Ingeniería Química. Blvd. M. García Barragán #1451, C.P. 44430, Guadalajara, Jalisco, México; jfasm@hotmail.com

[6] Centre de recherche Paul Pascal, UMR5031-Université de Bordeaux, 33600 Pessac, France; frederic.nallet@u-bordeaux.fr

[7] Universidad de Guadalajara, Departamento de Madera, Celulosa y Papel, Km. 15.5 Carretera Guadalajara-Nogales, Zapopan 45110, Jalisco, México; guillermo.tgonzalez@academicos.udg.mx

[8] Biomaterials Applications, 6 Rue Lesdiguières, 38000 Grenoble, France; marguerite.rinaudo38@gmail.com

* Correspondence: lourdes-monica.anaya@univ-rennes.fr

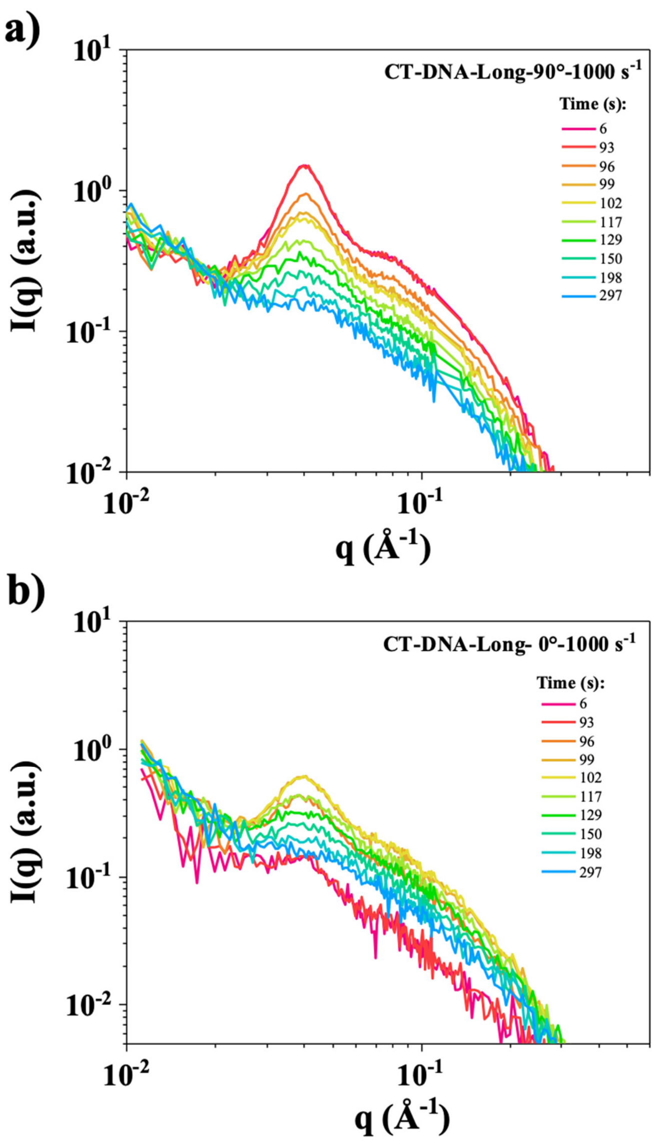

Figure S1. Intensity profiles from rheo-SAXS measurements at selected time intervals, illustrating the alignment during shear (1000 s^{-1}) and subsequent relaxation of DNA chains in the longitudinal configuration at a) 90° and b) 0°. CT-DNA solution was prepared at 15 mg/mL in water. Measurements were carried on at room temperature.
